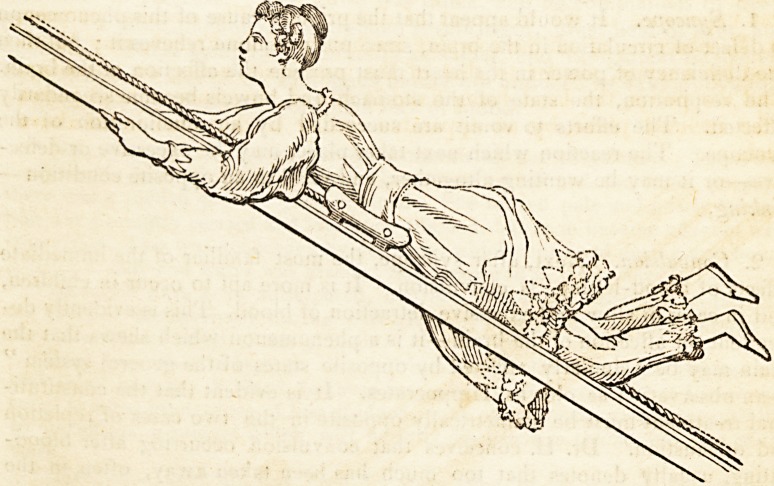# On the Mechanical Apparatus for Deformities

**Published:** 1830-07-01

**Authors:** 


### ON THE MECHANICAL APPARATUS FOR DEFORMITIES. By *L. J. Beale*, Surgeon.*

ALTHOUGH we have various monographs on particular varieties of spinal distortion, and many scattered papers on deformities of the limbs, yet there is no good and systematic description of these maladies in the English language. The task which Mr. Beale has undertaken is the collection and condensation of those observations which have been recorded by various authors, on the subject in question. In the performance of this task, the author appears to have used much diligence and exercised much judgment. The result is, unquestionably, a very useful elementary compilation, containing the pith or marrow of numerous heavy tomes, interspersed with judicious observations and practical hints. Such a work cannot, of course, bear a regular analysis ; but we have selected the twelfth chapter, on mechanical apparatus, for the subject of this short paper, in which will be found

* A Treatise on Deformities, exhibiting a concise View of the Nature and Treatment of the principal Distortions and Contractions of the Limbs, Joints, and Spine, &c. with plates and woodcuts. Octavo, 1830.

some curious particulars respecting the artificial means that have been resorted to by surgeons in former, as well as in present, times, for the cure or alleviation of this class of afflictions.

To describe a tenth part of the machinery invented for human distortions would require a large volume. Before the knowledge of anatomy was much diffused, the spine was considered by many as a single bone, and its curvatures were treated in the same way that a bent stick would be treated, when the object was to make it straight. Force was applied to the two extremities, to draw them into their proper position, and pressure was made on the prominent part. In our own days, a learned doctor, of manipulating celebrity, insisted that projections of the spine depended on dislocation of the vertebræ, and then the thumbing, stretching, and humbugging process came into vogue. The following woodcut, taken from Ambrose Parée, will shew that there is "nothing new under the sun." The centre figure is a rather flattering likeness of a modern spinewright, in the act of thumbing the dislocated vertebræ back into their places.

"It is fit to lay and stretch forth the patient upon a table, with his face downwards, and straitly to bind him about with towels under his arm-pits, and about the flanks and thighs. And then to draw and extend as much as we can, upwards and downwards, yet without violence; for unless such extension be made, restitution is not to be hoped for, by reason of the processes and hollowed cavities of the vertebræ, whereby, for the faster knitting, they mutually receive each other. Then must you lie with your hands on the extuberancies, and force in the prominent vertebræ. But if it cannot be thus restored, then it will be convenient to wrap two pieces of wood, of four fingers long, and one thick, more or less, in linen cloth, and so to apply one on each side of the dislocated vertebræ, and so with your hand to press them against the bunching forth vertebræ, until you force them back into their seats, just after the manner you see before delineated." 232.

We may readily conceive the dreadful consequences which must have occasionally ensued, in tearing asunder newly-formed anchyloses, by these dangerous extensions and endeavours to reduce supposed displacements of the vertebræ! During the last century all spinal distortions were mechanically treated, and collars, backboards, stays, spine-supporters, &c. were multiplied ad infinitum. In France, the celebrated Madame de Montmorençi lost her life by the stretching and screwing process employed to make her straight.

"The ill consequences of the mechanical treatment of deformities of the spine, frequently presented themselves. Slight distortions were rendered worse, and bad ones were not mended, and on the recommendation of Mr. Baynton it became the fashion to condemn all persons, who either had, or were threatened with spinal deformity, to an undeviating horizontal posture for months and years. This is excellent practice in many cases of diseased bone and cartilage, but in debilitated states of the muscular system, the evil will be increased by such total inaction. Frequent failures threw this system and the inclined plane, which was a modification of it, out of fashion." 234.

From the universal application of machinery in former times, we are now, Mr. Beale thinks, in danger of running into the opposite extreme—"of neglecting mechanical means and repose, and trusting too much to muscular exercise, gymnastics, and calisthenics." All the means enumerated are beneficial, when applied to the proper cases:—

"Various exercises in muscular debility, and in convalescence from diseases of the cartilages and bones: with the occasional use of spine-supporters in the intervals of repose and exercise: friction, manipulation and even pressure to lateral curvatures and projecting ribs: and undeviating rest in cases of caries." 235.

Machinery is used in the treatment of deformities on two principles—1st, to take off the superincumbent weight from bones, cartilages and ligaments, when these are diseased—2dly. to act on shortened, contracted, or rigid parts, and by extension to elongate them, or to bring them to their natural positions.

"Crutches are the simplest contrivances used on the first principle, when the lower limbs lose the power of supporting the weight of the body. The various backboards, collars, &c. used in curvatures of the spine are employed with the same view. Crutches have some advantage over corsets, and collars, inasmuch as they support the weight of the upper part of the body, without confining the muscles to total inaction. In walking, crutches should be of sufficient length to allow the extremity of the foot alone to touch the ground, and when attached to a seat, they should be high enough to raise the shoulders in the same manner as in the common mode of using them. The corsets employed in cases of distorted spine are occasionally useful, but much mischief has accrued from their indiscriminate employment. It must be obvious to every one, that where the primary source of distortion, is a debilitated state of the muscles, instruments which are employed to support the spine, to do, in fact, what the muscles should of themselves effect, so far from removing the deformity, will tend to the aggravation of the malady, they may conceal the defect but they never can cure it. Instruments of this kind are useful in slight cases of lateral curvature, and in convalescence from disease of the fibro-cartilages, in the intervals of exercise, during meals, and to relieve the irksomeness occasioned by long-continued recumbency." 236.

Our Gallic neighbours are almost mad, at the present period, in pursuit of machinery for the cure of distortions. Their MAISONS ORTHOPEDIQUES, vie with each other in the complexity of these machines. They not only stretch the spine, but apply mechanical means to act on the lateral contortions and inflections of the column. They keep up extension day and night, and their beds remind us of the bed of Procrustes, or the racks of the Inquisition. Here follows a specimen.

The above is taken from M. Delpech, a highly talented surgeon, who has great experience in the treatment of distortions. The essential purpose, Mr. B. observes, of all the complex machinery of the French, may be effected by a screw, similar to that of the tourniquet, acting on a bandage round the pelvis, the head being fixed in one of the common head-pieces of the inclined plane, by an apparatus similar to that seen in the above sketch.

The preceding wood-cut is copied from Delpech, and represents a contrivance by which exercise can be employed by convalescents from spinal diseases, as also in all cases where it is not yet proper to allow the weight of the body to press on the spine. By this apparatus a person can take exercise while in the recumbent position.

"The machine is supported on a basis, which moves on four rollers, in a grooved platform. The frame on which the cord is stretched, is connected with this basis by a pivot, by which the angle formed with the horizon may be altered. Below the axis there is a windlass in the frame which increases the tension of the cord. The car is mounted on the tense cord by two pullies, one anterior and one posterior: its sides cannot be supported on the sides of the frame, without impeding motion, which renders it necessary to maintain the equilibrium, by the action of the lower extremities." 242.

Below is a figure practising this exercise, and in the act of ascending. The cord is borne down by the weight of the body, the knees are pressed against the sides of the frame to maintain the equilibrium of the car, the body is raised by the exertion of the arms pulling at the side rails. This exercise obviously calls into play most of the muscles of the arms, chest, and spine, together with many of those of the lower limbs.

We are unable to pursue the subject any farther—the foregoing being indeed only a specimen of the ingenious author's work, which we recommend as a very useful epitome and compilation, in all orthopedic pursuits.

## Figures and Tables

**Figure f1:**
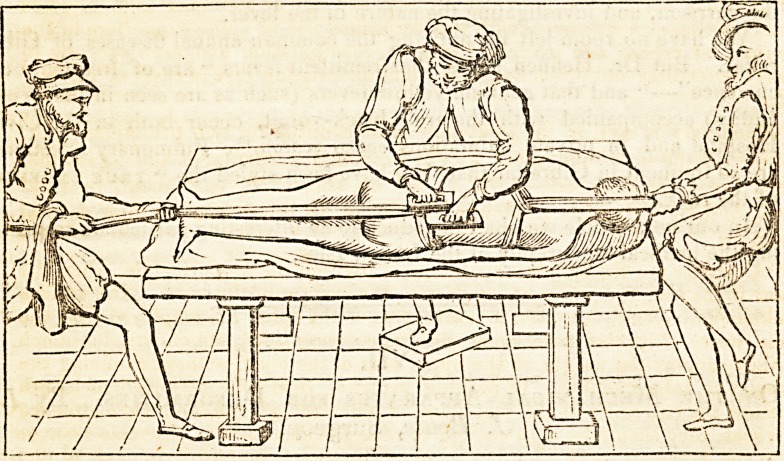


**Figure f2:**
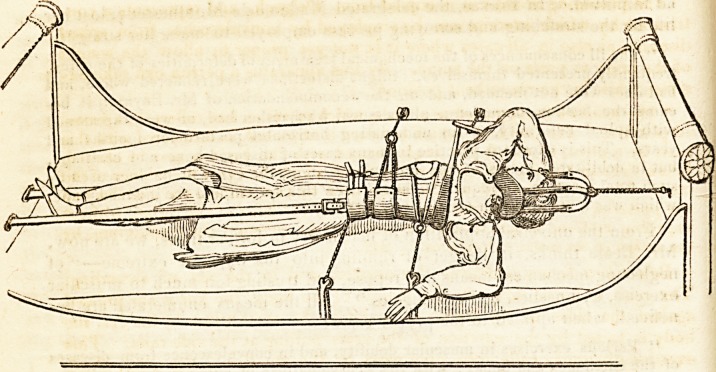


**Figure f3:**
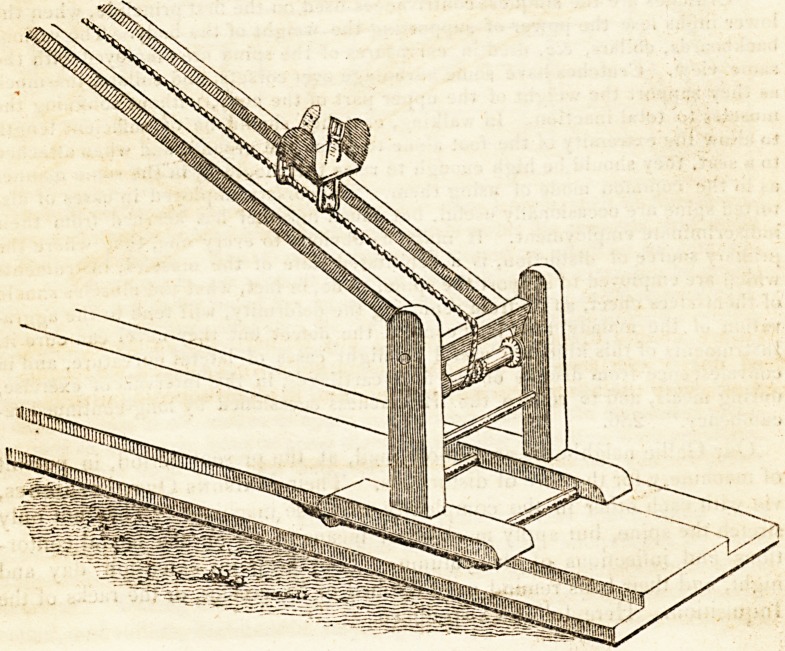


**Figure f4:**